# Holmium laser enucleation of prostate via perineal urethrostomy for the treatment of benign prostatic hyperplasia with mild long segment stenosis of the anterior urethra

**DOI:** 10.1007/s00423-026-03987-7

**Published:** 2026-03-04

**Authors:** Yunwu Hao, Lvwen Zhang, Wei Liu, Run Tao, Degang Chen

**Affiliations:** 1 Department of Urology, Lu’an Hospital Affiliated of Anhui Medical University, Lu’an, 237000 Anhui China; 2 Department of Urology, the First Affiliated Hospital of Anhui Medical University, Hefei, 230022 Anhui China

**Keywords:** Holmium laser enucleation of prostate, Perineal urethrostomy, Benign prostatic hyperplasia, Urethral stricture

## Abstract

**Purpose:**

To evaluate the efficacy of holmium laser enucleation of the prostate (HoLEP) via perineal urethrostomy. This procedure is used for treating benign prostatic hyperplasia (BPH) with mild long segment stenosis of the anterior urethra.

**Methods:**

The clinical data of 18 patients with BPH with mild long segment stenosis of the anterior urethra who underwent HoLEP via perineal urethrostomy from January 2023 to April 2024 were retrospectively analyzed. The patients’ baseline data, intraoperative conditions, and early postoperative complications were collected. The International Prostate Symptom Score (IPSS), quality of life (QOL), maximum urinary flow rate (Qmax), and postoperative residual urine (PVR) were compared before the operation, and at 1, 6 and 12 months postoperatively.

**Result:**

The operation time of 18 patients was 79.6 ± 13.2 min. Hemoglobin decreased by 12.7 ± 4.0 g/dl from before to after surgery. The length of urethral stricture was 3.7 ± 0.6 cm, including 11 patients with penile urethral stricture and 7 patients with bulbar urethral stricture. 5 cases (27.8%) experienced transient urinary incontinence, which resolved within 3 months after surgery. 1 case (5.6%) experienced perineal stoma stenosis and was resolved after three urethral dilations. Moreover, compared with preoperative, all patients showed significant improvement in Qmax, IPSS, QOL and PVR at 1, 6, and 12 months after surgery (*P* < 0.05). At 12 months after surgery, all patients had unobstructed urination and did not require secondary urethroplasty.

**Conclusion:**

HoLEP via perineal urethrostomy is a safe and effective alternative surgical option for treating BPH with mild long segment stenosis of the anterior urethra, which can overcome the limitations caused by urethral stricture and significantly improve patients’ lower urinary tract symptoms (LUTS). However, this procedure remains exploratory and remedial. Its effectiveness requires further validation through multicenter, large-sample studies with long-term follow-up.

## Introduction

Benign prostatic hyperplasia (BPH) is the most common cause of lower urinary tract symptoms (LUTS) in middle-aged and elderly men. Globally, more than half of men older than 60 years are affected by BPH [1]. Holmium laser enucleation of the prostate (HoLEP) is an effective treatment for BPH. Regarding the benefit/risk balance, HoLEP should be highlighted as the gold standard for the management of large-sized BPH [2]. However, BPH patients with urethral stricture present greater challenges for surgeons. According to the European Association of Urology (EAU) urethral stricture guidelines, urethral strictures with a lumen of 16Fr or greater was considered mild strictures without surgical intervention [3]. However, in patients with BPH and mild urethral stricture, traditional transurethral HoLEP is often halted because 24Fr or 26Fr resectoscopes cannot pass through the urethra.

At present, the conventional treatment strategies for patients with BPH with urethral stricture include open simple prostatectomy (OP), laparoscopic simple prostatectomy (LSP), robot assisted simple prostatectomy (RASP), etc. OP has a high risk of complications such as bleeding during the perioperative period, which currently limits its application [4]. However, with the development of laparoscopic technology, LSP and RASP have shown significant advantages in the treatment of patients with large-sized BPH [5, 6]. In 2018, a study first described the treatment of BPH using RASP via the extraperitoneal approach to preserve the urethra [7]. However, these surgical pathways are not transurethral approaches and are not routinely used for BPH treatment. RASP is more costly due to higher material expenses and the need for expensive robotic platforms. In addition, the treatment of BPH with mild urethral stricture also includes ultra-minimally invasive surgical techniques (uMISTs) that do not pass through the urethra, such as prostatic arterial embolization (PAE) and transperineal laser ablation (TPLA) [8]. However, compared to transurethral resection of the prostate (TURP), PAE has disadvantages in terms of functional outcomes (maximum rate of urinary flow and postvoid residual urine) [9].

In this study, we implemented HoLEP via perineal urethrostomy. This approach directly establishes the prostate surgical pathway by avoiding the narrow segment of the urethra. Meanwhile, this method combines HoLEP with optimization of the perineal urethrostomy pathway. Compared with other surgical methods, this surgical approach remains essentially unchanged and the operation is simple.

We established a channel from the perineal urethrostomy to the prostatic urethra to prevent rough sheath insertion, which can tear the urethral mucosa and worsen urethral stricture. Accordingly, this study aimed to evaluate the efficacy of HoLEP via perineal urethrostomy for treating BPH with mild long segment stenosis of the anterior urethra.

## Patients and methods

### General information

**Fig. 1:**
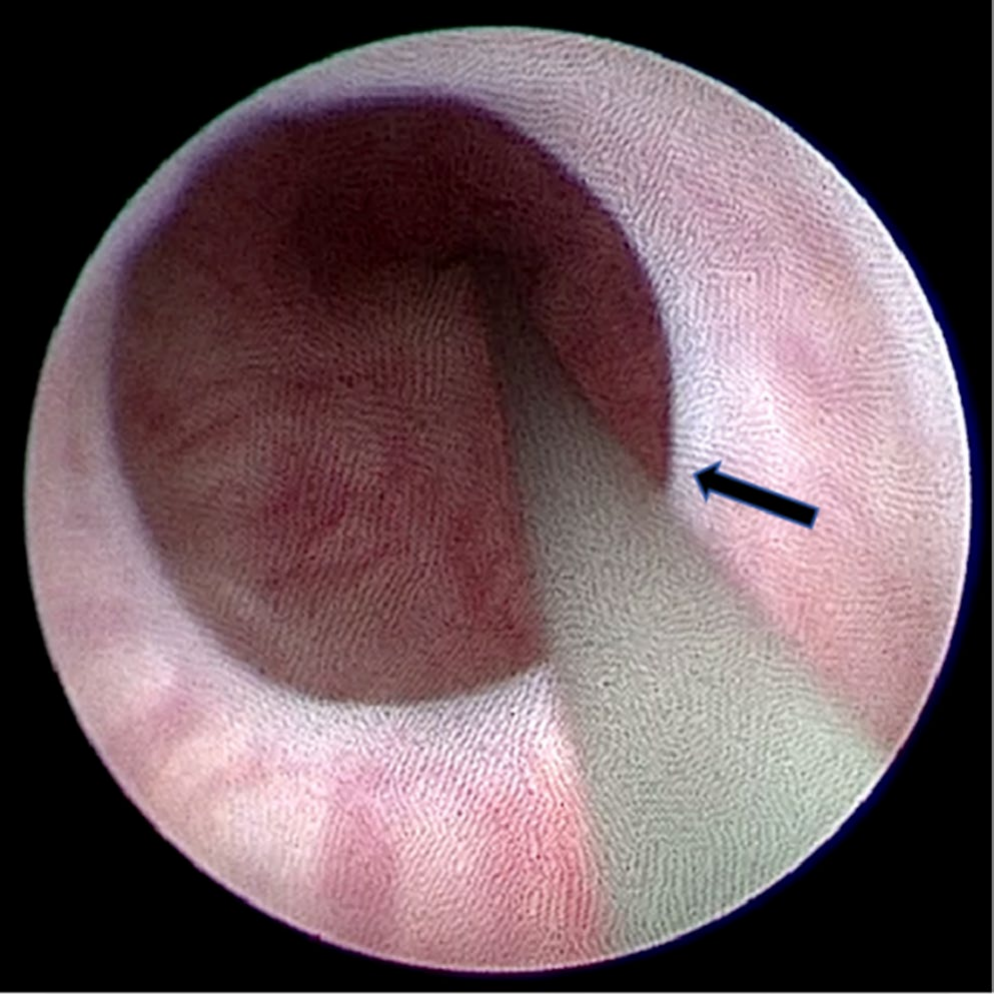
Cystoscopy examination for mild urethral stricture (arrow)

From January 2023 to April 2024, 463 patients with benign prostatic hyperplasia underwent HoLEP. During surgery, we found that some patients could successfully insert the 18Fr cystoscope but could not insert the 24Fr or 26Fr surgical sheath for prostate enucleation after urethral dilation (Fig. 1). We performed HoLEP via perineal urethrostomy on this group of patients. This study retrospectively analyzed the clinical data of 18 patients with BPH with mild long segment stenosis of the anterior urethra who underwent HoLEP via perineal urethrostomy. All surgical patients met the following inclusion and exclusion criteria. Inclusion criteria included: (1) Indications for surgery were based on Chinese urological disease diagnosis and treatment guidelines on BPH (2019); (2) According to EAU guidelines on urethral strictures, urethral stricture with a lumen of 16Fr or greater was considered mild stricture and asymptomatic incidental stricture [3]; (3) Urodynamic examination showed a significant increase in posterior urethral pressure, suggesting posterior urethral obstruction; (4) Intraoperative cystoscopy confirmed that the urethral stricture was located in the anterior urethra, with a length greater than 3.0 cm; (5) The urethra was able to pass through the 18Fr cystoscope, and the examination confirmed that prostate hyperplasia was compressing the posterior urethra. Exclusion criteria included: (1) Urodynamic examination showed that the function of bladder detrusor was severely impaired, and neurogenic bladder was considered; (2) Patients had indication for prostatic puncture; (3) Patients had a history of previous prostate surgery; (4) patients with severe organ dysfunction, such as cardiovascular disease, cerebrovascular disease, or pulmonary organ dysfunction.

The study design was approved by the ethics committee of Lu’an Hospital Affiliated to Anhui Medical University. All patients provided written informed consent to participate. This study was conducted in accordance with the Declaration of Helsinki and Good Clinical Practice (GCP) standards. The collected patient data included age, disease duration, body mass index (BMI), prostate volume, International Prostate Symptom Score (IPSS), Quality of Life (QoL), maximum urinary flow rate (Qmax), postoperative residual urine (PVR), prostate-specific antigen (PSA), and other relevant parameters.

### Surgical methods

**Fig. 2:**
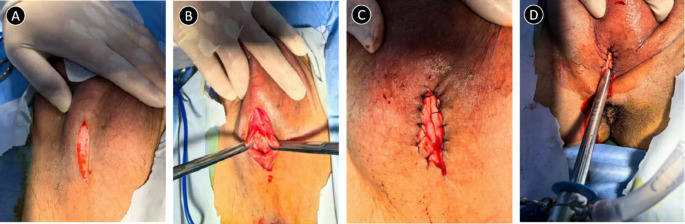
Surgical steps of perineal urethrostomy. **A** Starting from the skin on the proximal surface of the urethral stricture segment, make a longitudinal incision of approximately 3.0 cm towards the proximal end of the urethra; **B** Make an incision at the 6 o’clock position on the corpus cavernosum of the urethra; **C** Use 4 − 0 absorbable suture thread to intermittently suture the urethral mucosa onto the skin at the edge of the perineal incision; **D** HoLEP surgery is performed by placing an operation sheath through the perineal urethrostomy

The 96 W-SRM-H3B -Ho laser generator (Raykeen, Shanghai, China), with a 550 μm fibre and a 26 Fr resec-toscope sheath, was used during the HoLEP procedure. The energy settings were set at 2 J, 40 Hz for cutting and 0.8 J, 40 Hz for coagulation. Morcellation was performed with a YSB-III morcellator (HAWK, Guangzhou, China). The surgery was performed in two steps. The first step was perineal urethrostomy. If the sheath could not be inserted into the urethra after dilation, an 18Fr cystoscope was selected to examine the urethra and evaluate the location and length of the urethral stricture. After determining the proximal end of the urethral stricture, the skin on the proximal surface of the urethral stricture segment was used as the starting point for alongitudinal incision of about 3 cm toward the proximal urethra. The tissues of each layer were cut layer by layer, and the incision was made at the 6 o’clock position of the urethral cavernous body. A 4 − 0 absorbable suture was applied to intermittently suture the urethral mucosa to the skin at the margin of the perineal surface incision. In the second step, an operation sheath was placed through the perineal urethrostomy for the HoLEP procedure(Fig. 2).

**Fig. 3:**
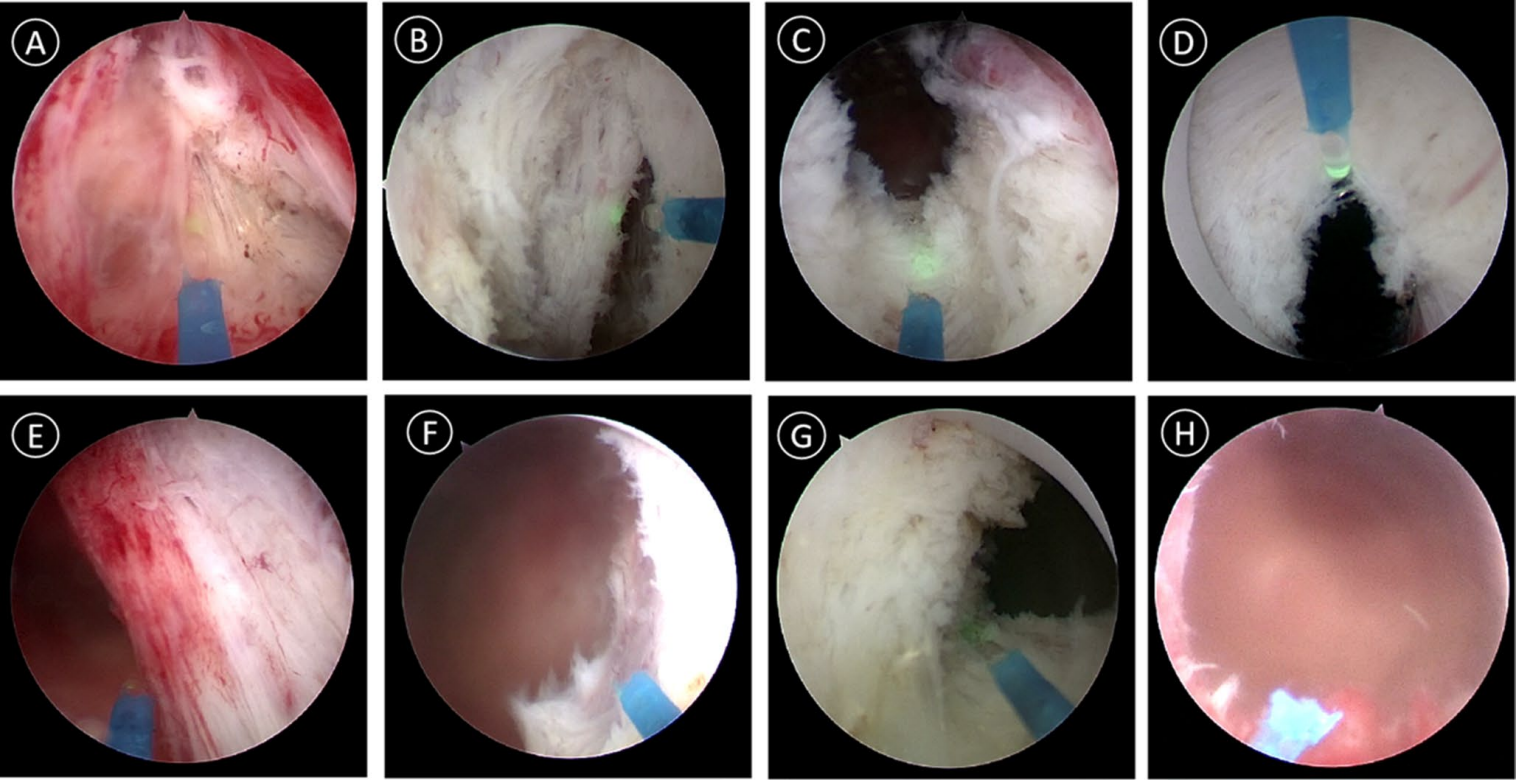
Surgical steps of HoLEP via perineal urethrostomy. **A** Make an inverted U-shaped laser incision anterior to the seminal vesicles. **B** Separate the bilateral lobes of the prostate along the plane of the prostatic capsule. **C** Perform laser incision starting from the 6 o’clock position of the bladder neck, extending to the plane of the prostatic capsule. **D** At the 12 o’clock position of the bladder neck, incise the urethral mucosa and extend the incision to the apex of the prostate gland, corresponding to approximately 1 o’clock in the left lobe and 11 o’clock in the right lobe. **E** Perform laser incision on the urethral mucosal strip between the external sphincter and the left lobe, and separate the left lobe from top to bottom along the surgical capsule plane. **F** Perform a laser incision of the bladder neck muscle fibers and completely remove the left lobe of the prostate gland and push into the bladder cavity. **G** Apply the same method to handle the right lobe. **H** View of the prostate gland fossa wound after surgery

All patients underwent HoLEP via perineal urethrostomy using the two-lobe or three-lobe technique. The two-lobe technique was the main approach, and the three-lobe technique was used for prostate enucleation in patients with BPH and middle lobe hyperplasia (Fig. 3). After surgery, a 20Fr three lumen catheter was inserted into the bladder through theperineal urethrostomy for continuous bladder irrigation. The balloon of Urinary catheter was filled with 30 ml of saline solution. All surgeries were performed by the same surgeon. The patient’s catheter was inserted and removed 4–7 days after surgery.

### Follow-up

The patients were followed up for complications including urinary retention, gross hematuria, bleeding, temporary urinary incontinence, urinary tract infection, testicular epididymitis andothers. At 1, 3, and 12 months after the operation, patients were reexamined by uroflowmetry, urethrography, or urethroscopy to evaluate surgical outcomes, including Qmax, IPSS, QOL, PVR, and PSA. Patients with a Qmax greater than 15 ml/s are considered to have unobstructed urination, which defines asuccessful surgery.

### Statistical analysis

Statistical analysis was performed using SPSS 23 (IBM Corp, Armonk, NY, USA). Paired samples t test was used for data with normal distribution, and the Mann Whitney U test was appliedfor data with abnormal distribution. *P* < 0.05 was considered statistically significant. Allexperimental data are displayed as the average value ± standard deviation.

## Results

The baseline demographic and clinical data of the 18 patients are summarized in Table 1. Among them, 14 cases (77.8%) used 5-alpha reductase inhibitors (5ARIs) alone or combined with alpha blockers, and 4 cases (22.2%) had indwelling catheters. The operation time of 18 patients was 79.6 ± 13.2 min. Hemoglobin decreased by 12.7 ± 4.0 g/dl on average after surgery compared to before surgery. The length of urethral stricture was 3.7 ± 0.6 cm, including 11 patients with penile urethral stricture and 7 patients with bulbar urethral stricture. Pathological diagnosis showed no prostate cancer. The intraoperative conditions and postoperative complications are shown in Table 2. 1 case (5.6%) had urinary retention after operation and retained catheterization again, and urination was normal after pulling out the catheter one week later. 5 cases (27.8%) experienced transient urinary incontinence, which disappeared within 3 months after surgery. 1 case (5.6%) experienced perineal stoma stenosis and was resolved after three urethral dilations. At each time point of follow-up, all patients showed significant improvements in Qmax, IPSS, QOL, and PVR compared with baseline (*P* < 0.05, Table 3). At 12 months after surgery, all patients had unobstructed urination and did not require secondary urethroplasty during follow-up.

**Table 1:** Baseline baseline demographic and clinical data in holep via perineal urethrostomy

Patient demographics (*n* = 18)	Results
Age (years)	70.7 ± 6.0
BMI (kg/m²)	26.4 ± 2.8
disease duration(year)	5.2 ± 2.3
5ARIs alone or combined with	
alpha blockersc (%)	14 (77.8%)
**Urogenital complications**	
with acute urinary retention (%)	4 (22.2%)
with gross hematuria (%)	5 (27.8%)
with bladder stones (%)	3 (16.7%)
with bladder trabeculae or chambers (%)	11 (61.1%)
with recurrent urinary tract infection (%)	7 (38.9%)
Prostate volume (ml)	62.4 ± 16.1
IPSS	25.3 ± 4.8
QoL	4.8 ± 0.8
Qmax (ml/s)	6.9 ± 2.9
PVR(ml)	158.7 ± 132
PSA(ng/ml)	4.0 ± 1.7

**Table 2:** Intraoperative conditions and early postoperative complications

Parameter	Results
Patients(n)	18
Operative time (min)	79.6 ± 13.2
Length of urethral stricture (cm)	3.7 ± 0.6
Prostate enucleated weight (grams)	50.8 ± 18.9
Hemoglobin decrease (g/dl)	12.7 ± 4.0
Capsular perforation (%)	1 (5.6%)
Prostate cancer (%)	0
Catheterization time (d)	4.8 ± 0.8
Urinary retention (%)	1 (5.6%)
Postoperative Hematuria (%)	4 (22.2%)
Hemorrhage (%)	1 (5.6%)
Transient incontinence (%)	5 (27.8%)
Urinary tract infections (%)	2 (11.1%)
Testicular epididymitis (%)	0
Urethral stricture (%)	1 (5.6%)

**Table 3:** Functional outcomes after holep via perineal urethrostomy

	Baseline	1 mo	6 mo	12 mo	Baseline	Baseline	Baseline
					VS. 1 mo	VS. 6 mo	VS. 12 mo
Q_max_(mL/s)^a^	6.9 ± 2.9	17.9 ± 4.2	19.1 ± 4.1	21.7 ± 2.1	< 0.0001	< 0.0001	< 0.0001
IPSS^a^	25.3 ± 4.8	7.7 ± 2.9	6.7 ± 2.8	6.0 ± 0.7	< 0.0001	< 0.0001	< 0.0001
QoL^b^	4.8 ± 0.8	1.7 ± 0.9	1.1 ± 0.8	0.8 ± 0.3	< 0.0001	< 0.0001	< 0.0001
PVR(mL)^b^	158.7 ± 132	6.6 ± 3.4	6.2 ± 2.5	5.6 ± 2.1	< 0.0001	< 0.0001	< 0.0001
PSA(ng/mL)^a^	4.0 ± 1.7	1.6 ± 0.6	1.2 ± 0.5	1.2 ± 0.2	< 0.0001	< 0.0001	< 0.0001

^a^Normally distributed variable data analyzed with independent-sample t test

^b^Non-normally distributed variable data analyzed with the Mann-Whitney U test

## Discussion

The management of BPH with anterior urethral stricture is a thorny problem for urologists. During the operation of HoLEP, the sheath usually cannot pass through the urethral stricture. Violent operation easily leads to urethral injury and postoperative urethral stricture. In this study, HoLEP via perineal urethrostomy is shown to be a safe and effective alternative surgical option for treating BPH with mild anterior urethral stricture. This approach can significantly improve the LUTS of BPH patients. These results are consistent with previous studies [10–12]. While retaining the minimally invasive advantage of HoLEP, this operation effectively circumvents the limitation of anterior urethral stricture and provides a safe and effective solution for BPH patients with urethral stricture.

Transurethral surgery is the most commonly performed procedure for BPH, including TURP, HoLEP, thulium laser enucleation of the prostate (ThuLEP), and greenlight laser enucleation of the prostate (GreenLEP) [13]. Previous studies have shown that HoLEP is superior to conventional transurethral prostate enucleation techniques [14, 15]. In addition, it does not cause additional trauma related to the surgical pathway and is considered to be the most likely gold standard for the treatment of BPH [16]. The prerequisite for HoLEP is transurethral access. However, the surgical sheath cannot pass through a narrow urethra, making HoLEP unsuitable for BPH patients with urethral stricture. Recent studies have shown that the miniaturized holmium laser enucleation of the prostate (MiLEP) is effective in treating BPH, reducing early postoperative stress urinary incontinence and thereby shortening the recovery period [17, 18]. However, the operating sheaths of 22Fr and 18.5Fr still cannot pass through 16-18Fr urethral strictures, making it impossible to treat BPH with concomitant 16-18Fr anterior urethral strictures. OP and minimally invasive simple prostatectomy (MISP) are usually applied to patients with BPH and mild anterior urethral stricture. However, these procedures cause greater trauma compared with endoscopic enucleation of the prostate (EEP), and they also require a longer duration of urinary catheter indwelling [19]. In addition to surgical options, other methods including transrectal MRI or ultrasound-guided local laser ablation for the treatment of LUTS caused by BPH have been proved to be safe and effective [20–22]. However these methods have not been carried out in the majority of medical centers and do not belong to the mainstream methods for the treatment of BPH. Mild urethral stricture is often discovered during surgery, and patients usually find it difficult to accept switching to laparoscopic prostatectomy at that time. Meanwhile, the nursing staff in the operating room also require more effort and time to prepare the equipment. For most patients, the surgery can be stopped midway, and a second-stage laparoscopic prostatectomy or alternative procedure can be scheduled later. In this study, HoLEP via perineal urethrostomy was applied to overcome these shortcomings. The surgical method itself remained unchanged. HoLEP via perineal urethrostomy can be successfully performed by inserting the surgical sheath, which is achieved by optimizing the surgical approach and avoiding urethral stricture.

At present, the mainstream surgical techniques of HoLEP include enbloc, two-lobe technique and three-lobe technique. Each technique has its own advantages and disadvantages [23, 24]. However, enbloc and two-lobe enucleation are significantly faster in terms of enucleation time, overall operation time, and speed compared to the three-lobe technique [25]. Taking the two-lobe technique as an example, we have made technical improvements to address difficultions encountered during laser enucleation of the prostate. First, when the enucleation of the middle lobe reaches the bladder neck, there is a risk of inadvertently entering the posterior side of the bladder. To address this difficulty, before enucleating the middle lobe and reaching the bladder neck, we first cut the bladder neck and urethral mucosa anterogradely at 6 o’clock to the proximal end of the seminal vesicle. Then, we fused the cut bladder neck with the surgical capsule. In this way, the opened bladder neck is used as a marker to effectively avoid enucleation into the posterior side of the bladder. Second, long-term follow-up after HoLEP indicates that urinary incontinence is a major concern [26]. To avoid postoperative urinary incontinence, we reserved part of the urethral mucosa of the prostate apex at the 12 o’clock position. We opened the urethral mucosa from the bladder neck at 12 o’clock to the surgical capsule plane, then cut it anterogradely along the surgical capsule plane to the tip of the prostate (right at 11 o’clock, left at 1 o’clock), forming an inverted Y-shaped groove. Fully separating the bilateral lobes can not only preserve the urethral mucosa of the prostate apex but also entirely remove the prostate gland. This approach also helps prevent the occurrence of urethral stricture. Early detachment of the urethral mucosa at the tip of the prostate lateral lobe reduces mechanical traction on the urethral sphincter, which is beneficial for the protection of urinary control function. Previous studies have demonstrated that preservation of the urethral mucosa at the prostate apex effectively reduces the incidence of postoperative urinary incontinence [27]. Third, rapid enucleation of the prostate is important. Relevant studies have shown that HoLEP offers shorter of operation times compared to LSP and OP [28]. In this study, we optimized the HoLEP procedure. Taking the left lobe as an example, the lateral lobe of the prostate can be rapidly enucleated by fusing the surgical capsule plane at 5 o’clock and 1 o’clock, after detaching the urethral mucosa at the tip of the lateral lobe, and then separating from top to bottom along the surgical capsule plane. This method offers simple operation, simplified steps, reduced bleeding, and rapid completion of the procedure.

After completing HoLEP, we opted for a two-stage closure of the perineal fistula. This decision was mainly based on the following reasons. Firstly, urethral reconstruction for anterior urethral stricture requires patch techniques, such as penile scrotal skin flaps or oral mucosa grafts, to enlarge the urethral cavity. This leads to prolonged surgical time [29, 30]. Secondly, complications such as hematuria and postoperative bleeding may occur after HoLEP, and simultaneous urethral reconstruction surgery may increase the risk of surgical failure [31, 32]. Therefore, performing urethral reconstruction surgery in the second stage is more reasonable.

Perineal urethrostomy can provide an excellent, durable success rate with high levels of patient satisfaction [33, 34]. Our study reached a similar conclusion. All patients avoided secondary urethroplasty because they were satisfied with smooth urination and did not mind squatting to urinate. This study mainly utilized the concept of using the perineal urethrostomy tract as a surgial access route to perform HoLEP, thereby combining the advantages of the two surgical methods. Therefore, HoLEP via perineal urethrostomy is a safe and effective alternative surgical option for treating benign prostatic hyperplasia with mild anterior urethral stricture, especially in cases where most medical centers have inadequate surgical equipment.

## Conclusion

HoLEP via perineal urethrostomy is a safe and effective alternative surgical option for treating BPH with mild long segment stenosis of the anterior urethra, which can overcome the limitations caused by urethral stricture and significantly improve patients’ lower urinary tract symptoms (LUTS). However, this procedure remains exploratory and remedial. Its effectiveness requires further validation through multicenter, large-sample studies with long-term follow-up.

## Data Availability

The datasets used and/or analysed during the current study are available from the corresponding author on reasonable request.

## Abbreviations

HoLEP: holmium laser enucleation of the prostate
BPH: benign prostatic hyperplasia
EAU: European Association of Urology
IPSS: International Prostate Symptom Score
QOL: quality of life
Qmax: maximum urinary flow rate
PVR: postoperative residual urine
LUTS: lower urinary tract symptoms
OP: open simple prostatectomy
LSP: laparoscopic simple prostatectomy
RASP: robot assisted simple prostatectomy
uMISTs: ultra-minimally invasive surgical techniques
TIND: temporary implantable nitinol device
PAE: prostatic artery embolization
TPLA: transperineal laser ablation
BMI: body mass index
PSA: prostate-specific antigen
TURP: transurethral resection of the prostate
ThuLEP: thulium laser enucleation of the prostate
GreenLEP: greenlight laser enucleation of the prostate
MISP: minimally invasive simple prostatectomy
EEP: endoscopic enucleation of the prostate
